# A thermosensitive gel based on *w1/o/w2* multiple microemulsions for the vaginal delivery of small nucleic acid

**DOI:** 10.1080/10717544.2019.1568622

**Published:** 2019-03-01

**Authors:** Jiu Wang, Yajing Wang, Ziqiang Wang, Fan Wang, Jie He, Xiaoyun Yang, Weidong Xie, Ying Liu, Yaou Zhang

**Affiliations:** a School of Pharmaceutical Sciences, Hubei University of Medicine, Shiyan, Hubei, China;; b Division of Life Science, Key Lab in Healthy Science and Technology, Graduate School at Shenzhen, Tsinghua University, Shenzhen, China;; c Department of Pharmacy, School of Pharmaceutical Engineering and Life Science, Changzhou University, Changzhou, Jiangsu, China;; d School of Basic Medical Sciences, Hubei University of Medicine, Shiyan, Hubei, China

**Keywords:** Small interfering RNA (siRNA), multiple microemulsions (MMEs), low energy emulsification (LEE), thermosensitive gel, vaginal delivery

## Abstract

The present study aims at designing a thermosensitive gel prepared from *w1/o/w2* multiple microemulsions (MMEs) for the vaginal delivery of siRNA. The *w1/o/w2* MMEs were prepared by two-step emulsifications: the first step was to prepare primary emulsions (*w1/o*) by low energy emulsification (LEE); the second step was to obtain stable *w1/o/w2* MMEs by self-emulsifying. An extensive formulation optimization process was undertaken. The final *w1/o/w2* MMEs could be formed in ddH_2_O, phosphate buffer solution (PBS, pH 7.4) and 1640 culture media with diameter size about 166.5 ± 13.1, 271.0 ± 11.1 and 278.7 ± 12.1 nm respectively. The release rates of siRNA from solutions, MMEs and MMEs-gels were completed within 2 h, 6 h and13 h respectively. The transfection efficiency of MMEs was confirmed both *in vitro* and *in vivo*. The relative target gene expressions of MMEs were 0.07 ± 0.05% vs. 0.37 ± 0.06% in *Hela* cells against Lipofectamine2000® and 1.88% ± 0.00% vs. 9.65% ± 0.02% in mouse vaginal mucosa against PEI. Good biocompatibility of MMEs was verified by cytotoxicity and pathological studies. Overall, our results indicated the potential of the MMEs-gel system for the vaginal delivery of siRNA.

## Introduction

RNAi therapy utilizes short interfering RNA (siRNA) duplexes, usually composed of 19–25 nucleotides to afford an excellent therapeutic opportunity by the modulation of target gene expression for the prevention of many diseases. However, for translating RNAi from an experimental approach to a clinically viable therapeutic strategy that can benefit patients, there is still a critical need for a safe and effective delivery system that can package the labile payload, afford stability during transport, permeability through tissue and cell membrane barriers as well as cytosolic bioavailability for efficient gene silencing (Vicentini et al., 2013; Videira et al., 2014).

The identification of siRNA targets for cervical cancer (E6, E7 and Grb10), human immunodeficiency virus (HIV) infections (CCR5) or HSV-2 infections (UL-29ANDUL-27) have increased the interest in the development of vaginal delivery systems for the treatment and/or prevention of infectious diseases (Wu et al., 2011; Vicentini et al., 2013; Gu et al., 2015). Vaginal delivery of siRNA could lead to the silencing of endogenous causative genes in the genital tract and protect against challenge from infectious disease. Vaginal route has advantages over other routes such as large area, rich blood supply, accessibility and circumvention of first-pass hepatic clearance (Woolfson et al., 2000; Kammona & Kiparissides, 2012; Vanić & Škalko-Basnet, 2013; Yang et al., 2013). Since viral vectors are associated with several disadvantages making it a huge concern for broad applications despite its efficiency in delivering siRNA intracellularly, non-viral gene delivery systems are rapidly emerging as alternative carriers, because of their ease of production and nonimmunogenicity, such as liposomes (Schafer et al., 2010; Taruttis et al., 2014; Yu et al., 2018), polycationic polymers (Zheng et al., 2012; Du et al., 2014; Wu et al., 2018), conjugates (Wu et al., 2018), polymeric nanoparticles (Mishra et al., 2012; Chen et al., 2018) and so on.

Lipid based siRNA delivery systems such as lipoplexes composed of cationic lipid and anionic siRNA or liposome have been evaluated extensively for siRNA delivery (Schafer et al., 2010; Geusens et al., 2011; Ho et al., 2013; Lechanteur et al., 2017; Wu et al., 2018). The cationic siRNA lipoplexes facilitated the electrostatic interaction with anionic cell membrane and contributed to the intracellular transport of therapeutic siRNA. Palliser et al. (2006) successfully employed Oligofectamine^®^ to deliver a siRNA-based microbicide into mice genital tract to prevent the infection of HSV-2. Later on Wu et al. (2009) prepared cholesterol-conjugated-siRNAs lipid to silence gene expression in the vagina, which provided sustained protection against viral transmission. Cholesterol was coupled to the 3-end sense strand of siRNA to form a neutral transfection complex, which could deliver siRNA intracellularly without interrupting cell integrity or eliciting an inflammatory response. In addition, Wu et al. (2011) developed an alginate scaffold system containing muco-inert PEGylated lipoplexes to provide a sustained vaginal presence of lipoplexes *in vivo* and facilitate the delivery of siRNA/oligonucleotides into the vaginal epithelium. The presence of alginate increases their uptake into the vaginal epithelium compared to existing transfection systems in mice. A significant knockdown of Lamin A/C level was also observed in vaginal tissues. Gu et al. (2015) fabricated PEG-functionalized PLGA/PEI/siRNA nanoparticles (NP), which were decorated with anti-HLA-DR antibody (siRNA-NP-Ab) for targeting delivery to HLA-DR + dendritic cells (DCs) and homogeneously dispersed in a biodegradable film consisting of polyvinyl alcohol (PVA) and lambda-carrageenan. The siRNA-NP-Ab-loaded film (siRNA-NP-Ab-film) was transparent, displayed suitable physicochemical properties, and was noncytotoxic. Targeting activity was evaluated in a mucosal coculture model consisting of a vaginal epithelial monolayer (VK2/E6E7 cells) and differentiated KG-1 cells (HLA-DR + DCs).

Multiple emulsions, such as water-in-oil-in water (*w1/o/w2*), consist of primary water in oil (*w1/o*) emulsions, which are themselves dispersed in external water, producing the *w1/o/w2* multiple emulsions. The main advantages of *w1/o/w2* included (1) enhancing permeability and absorption of hydrophilic compounds such as insulin via the enteric route; (2) versatile delivery of both of hydrophilic and hydrophobic drugs within internal water phase and interlayer oil phase simultaneously and (3) improving the stability of siRNA by encapsulation into internal *w1*. However, the application of multiple emulsions was limited by their poor stability due to thermodynamic instability. The design and development of multiple microemulsions (MMEs) with better stability and permeability by the delicate selection of formulation and preparation techniques shows promise (Garti & Bisperink, 1998; Schuch et al., 2014). The double emulsion technique has been extensively used for the encapsulation of hydrophilic compounds including protein and peptides. Encapsulation of these compounds could prevent their degradation, control the rate and extent of release and enhances the loading efficiency.

However, liquid formulations are inappropriate for sustained drug release due to their short residence time in the vaginal cavity; while conventional semisolids suffer from relatively low patient acceptability and poor retention because the self-cleansing mechanisms within the vagina. Thermosensitive gels possessing a reversible sol-gel transition behavior in aqueous media have been utilized for vaginal delivery of various drugs with the advantages of prolonging drug release and administration convenience by many researchers (Almomen et al., 2015; Saini et al., 2016; Ci et al., 2017). In the present study, we managed to prepare MMEs based thermosensitive gels carrying siRNA for the vaginal delivery, which could lead to efficient target gene silencing in the genital tract and protect against challenge from HPV infectious disease. Low energy emulsification (Lin, 1976; 1978; Solans & Sol, 2012) was employed to prepare primary emulsions (*w1/o*) to protect siRNA from degradation. The transfection efficiency of final MMEs carrying siRNA was compared with Lipofectamine2000^®^ (Lipo2000) *in vitro* and PEI *in vivo* respectively. The result verified that final *w1/o/w2* MMEs carrying siRNA could permeate into vaginal mucosa and inhibit the expression of target gene *in vivo*. More importantly, the cytotoxicity of MMEs was almost invisible in the formulation amount. To the best of our knowledge, the application of MMEs prepared by self-emulsification for the siRNA vaginal delivery was less reported.

## 1. Methods

### 1.1 Materials

Span80, Span85, Tween80, Soybean oil, Squalene (Aladdin, China). Lecithin (A.V.T. Pharmaceutical, Shanghai). Oleic acid, medium chain triglyceride (MCT) were provided by GATTEFOSSé (Paris, France). RH40, Polyoxyl 35 castor oil (Cremophor®EL) were purchased from BASF (Ludwigshafen, Germany). Glycerol and Propylene glycol were provided by Fagron Nordic A/S (Rotterdam, The Netherlands). All other reagents were of pharmaceutical grade. Reagents for cell culture were provided by Gibco (Montpellier, France).

A 21-base pair siRNA oligonucleoside duplex for silencing B2M gene was synthesized by the help of ‘siRNA Explorer’ and dissolved in diethylpyrocarbonate (DEPC)-treated water before use. The gene sequence was confirmed by HPLC-MS and gel electrophoresis comprehensively. The gene silencing efficiency was verified *in vitro*. Jet-PEI was provided by Polyplus Company. Lipo2000 was provided by Invitrogen Company.

NIH female mice (8 weeks, 20 ∼ 28 g) in estrous phase were provided by Guangdong Medical Experimental Animal Center (China). The study protocol was reviewed and approved by the Animal Welfare and Ethics Committee of Tsinghua University.

### 1.2 Preparation of Self-emulsifying MMEs

MMEs were prepared using a two-step emulsification process: the first step to form the primary emulsions (*w1/o*) and the second step to prepare self-emulsifying multiple emulsions, which could form MMEs (*w1/o/w2*) in water phase under mild stirring. An extensive formulation optimization process was undertaken (Garti & Bisperink, 1998; Solans et al., 2005; Solans & Sol, 2012).

#### 1.2.1 Primary *w1/o* emulsions

In the first step, primary *w1/o* microemulsions were prepared by Phase Inversion Emulsification Method (PIEM). Briefly, oil phase was added drop-by-drop into the internal water phase (*w1*) with equal amount under stirring at 1000 rpm on the ice bath for 8 min to ensure thorough mixing. *w1* was composed of PBS and lipophilic surfactants (LS). Then the temperature was increased to 60 °C for 5 min. After that the remained oil phase was added into the mixtures. The solutions were homogenized at 8000 rpm for 3 min to form primary *w1/o* microemulsions.

At a constant 50% *w1* fraction, 13–14% LS fraction (mixture of Span80 and RH40 with volume ratio varied from 10:1 to 10:8), the effects of different oils and co-surfactants on the formation of single *w1/o* emulsion were studied. MCT, soybean oil, squalene and oleic acid were selected as oil phase; while lecithin, glycerol and propylene glycol were selected as co-surfactants.

#### 1.2.2 Final *w1/o/w2* MMEs

Secondly, the previous *w1/o* microemulsion was mixed with hydrophilic surfactant (HS), with the formulation ratio to obtain self-emulsifying MMEs. The *w1/o/w2 MMEs* would be formed under mild stirring. MMEs carrying siRNA were prepared similarly with the addition of siRNA in *w1* phase in the first step (MMEs-siRNA).

Based on the preliminary results, the influence of HS and/or co-HS on the formation of a clear, transparent isotropic formulation, the so-called multiple microemulsions, was fully investigated. Various formulations of coupled HS/co-HS were prepared according to L16 (4^2^) orthogonal design (Table 1). Self-emulsifying efficiency (Y1, score), productivity (Y2, %), centrifugal stability (Y3) were taken as index. Y1 was evaluated by visual observation, which could be described as, weigh 1 g samples, the add 5 mL PBS under 37 °C with stirring. Observe the appearance of MMEs formed visually and ranked by the following rules:

**Table 1.** The levels of experimental factors.

	HS	co-HS
1	RH40	Glycerol
2	EL30	Lecithin
3	RH40/Span80 = 9:1	Propylene glycol
4	EL30/Span80 = 9:1	/

- Form clear or mild blue opalescence microemulsion, give 5 score;
- Form thick milky emulsion with blue opalescence quickly, give 4 score;
- Form thick milky emulsion quickly without blue opalescence, give 3 score;
- Form thick milky emulsion slowly without blue opalescence, give 2 score;
- Degree of emulsification is poor, surface with large oil droplets, give 1 score.

The productivity (Y2) was calculated by the following formula (Videira et al., 2014) after determination of conductivity.

(1) $$\text{Productivity }(\%)\;=100\times \;[S_{1}-A\times (S_{2}-S_{3})÷B]÷S_{1}$$

In which, S_1_, S_2_ and S_3_ is the conductivity of 1 gL^−1^ NaCl solution, MMEs prepared by 1 gL^−1^ NaCL solution and blank MMEs respectively; A is the total volume of formed emulsions (mL); B is the volume of internal water phase (mL).

Centrifugal stability (Y3) was evaluated by the following method. Firstly, the final MMEs were centrifuged at 3000 rpm for 5 min and observed. If it couldn’t keep clear and transparent, give 1 score; or centrifuged at 5000 rpm for 5 min and observed again. If it couldn’t keep clear and transparent, give 3 score, or give 5 score. The overall score is weighted by Y1 30%, Y2 40% and Y3 30%.

### 1.3 Characterization of MMEs

Weigh 1 g samples, the add 5 mL dH2O, 0.1 M pH 7.4 phosphate buffer solution(PBS) and 1640 culture media under 37 °C with stirring respectively. The resultant MMEs were characterized by particle size, surface charge (ZetaSizerNano ZS, Malvern Instruments, UK) and morphology（TEM, TecnaiG2 F30, FEI, USA).

MMEs carrying siRNA (siRNA-MMEs) were prepared similarly with the addition of siRNA in *w1* phase in the first step described in section ‘1.2’. siRNA-MMEs-gels were prepared by mixing siRNA-MMEs with ‘thermosensitive gels’ (provided by South China pharmaceutical company with Gelation temperature at 34.7 ± 0.2 °C and employed in following experiments) with volume ratio of 1:5.

The release of siRNA from siRNA-MMEs and siRNA-MMEs-gels were determined by using a modified dialysis method in comparison of siRNA in RNAase-free solution. SiRNA-MMEs, siRNA-MMEs-gels and siRNA solutions were sealed in cellulose ester membrane dialysis bags (cut off 30,000 Da) respectively. The siRNA concentration was determined as 400 μg/μL in all the formulations. The formulations loaded dialysis bags were then immersed in the dissolution media (0.1 M pH 7.4 PBS) contained in a beaker and maintained at 37 °C. At specific intervals, aliquot samples were taken from the dissolution medium and replenished with PBS. The samples were analyzed by the absorbance of siRNA at 260 nm. The accumulative release percent of siRNA was calculated.

### 1.4 Cellular uptake studies and cytotoxicity evaluations of MMEs

#### 1.4.1 Cell culture

*Hela* cells derived from human cervical carcinoma were cultured in a DMEM medium with 100 U/mL penicillin, 100 μg/mL streptomycin, and 10% (*v: v*) FBS at 37 °C, 5% CO_2_. The cells were subcultivated approximately every 3 days at 80% confluence using 0.25% (*w: v*) trypsin at a split ratio of 1:5.

#### 1.4.2 Cell viability

*In vitro* anti-proliferation of siRNA-MMEs against *Hela* cells was estimated by WST-8 method (Latiff et al., 2015). The cells were seeded at a density of 2*10^4^ cells/mL in a 96-well plate and cultured for 24 h. The cells were exposed to the culture medium (200 μL) containing different concentration MMEs (0.5, 1, 2, 3, 4, 5 μL) for 24, 48 and 72 h, followed by adding CCK-8 kit. After 4 h, the medium was removed, and the cells were stained with CCK-8 reagent. The absorbance of each well was read on a microplate reader (Thermo Scientific Varioskan flash spectral scanning multimode reader) at a test wavelength of 450 nm. The half maximal inhibitory concentration (IC50) was calculated using LOGIT method.

$$\text{Inhibition }(\%)=\frac{\text{Absorbance}\;of\;\text{negative}\;\text{group}\;-\;\text{absorbance}\;of\;\text{test}\;\text{group}}{\text{Absorbance}\;of\;\text{negative}\;\text{group}}\times 100\%$$

#### 1.4.3 Cellular uptake studies

To evaluate the cellular uptake efficiency of siRNA-MMEs, siRNA was labeled by FAM firstly (FAM-siRNA). MMEs carrying FAM-siRNA (FAM-siRNA-MMEs) were prepared similarly with the addition of FAM-siRNA in *w1* phase in the first step. Then 1 μL FAM-siRNA-MMEs was added into 100 μL serum-free medium and employed as test group. FAM-siRNA (50 μL, 0.27 μg/μL) and Lipo2000 (50 μL Invitrogen) were diluted by serum-free medium into 100 μL and incubated for 5 min at room temperature (R.T.), respectively. Then the above mentioned two solutions were mixed together with equal amount followed by incubating for 20 min at R.T. and employed as positive control (FAM-siRNA-Lipo2000). FAM-siRNA-MMEs and FAM-siRNA-Lipo2000 were mixed with ‘thermosensitive gels’ in volume ratio 1:5 to 1:3, respectively.

The *Hela* cells in logarithmic growth phase were transfected with FAM-siRNA, FAM-siRNA-MMEs, FAM-siRNA-MMEs-gels, FAM-siRNA-Lipo2000 and FAM-siRNA-Lipo2000-gels for 6 h in 96 wells. The medium was replaced by DMEM-10%FBS culture medium for next 24 h, followed by detecting the fluorescence intensity using fluorescence microscope.

### 1.5 *In vitro* gene silencing

The efficacy of gene silencing of siRNA-MMEs was investigated by detecting the expression of target gene. Random oligonucleotide-Lipo2000 and siRNA-Lipo2000 were prepared by the method described in ‘1.4.3’ section and employed as negative and positive controls respectively.

The *Hela* cells in logarithmic growth phase were then incubated with siRNA-Lipo2000 and siRNA-MMEs for 6 h, while random oligonucleotide for 24 h, followed by detecting the expression of target gene by Q-RT-PCR, respectively.

### 1.6 *In vivo* gene silencing

Random oligonucleotide-PEI, siRNA-PEI, and siRNA-MMEs were prepared by the method described in ‘1.4.3’ section and employed as negative, positive and test group. The above mentioned 3 kinds of formulations were then mixed with ‘thermosensitive gels’ in volume ratio 1:3 ∼ 1:5 respectively. The final concentration of siB2M is designed as 127.22 μg/ml.

A randomized group division study, based on the NIH female mice, was conducted to investigate the efficacy of vaginal siRNA delivery. The mice were anesthetized with an intraperitoneal injection of Ulatan. Different formulations (60–65 μL) were administered to the vagina respectively. The mice were inverted for 3–5 min and shaked gently to make the formulation gelation. The formulations were administered again. Twenty four hours later, the mice were sacrificed, and the vaginas were flush with sterilized and RNAase PBS for 1 ∼ 3 times (60 μL/time). Then, the vaginas were excised and everted. The vaginal openings were tied while cervix was immersed in Trizolsolutions (Invitrogen) to extract total RNA. The expressions of target gene and siRNA were detected by Q-RT-PCR.

### 1.7 Histological analyses

A randomized group division study, based on the NIH female mice, was conducted to evaluate if the blank thermosensitive hydrogels would lead to the injury of vaginal mucosa. The mice were anesthetized with an intraperitoneal injection of sodium Ulatan. MMEs gels, PEI-gels and PBS-gels (60 μL) were administered to the vagina respectively. The mice were administered again 24 h later. The mice were sacrificed, and the vaginas were flush with sterilized PBS for 1–3 times (60 μL/time) 48 h later. Then, the vaginas were excised and fixed in 4.0% (*w/v*) paraformaldehyde overnight, and then embedded in paraffin. The paraffin-embedded vagina tissues were cut with a thickness of 6.0 μm and stained with hematoxylin and eosin(H&E) to assess histological alteration by microscope (Nikon TE2000U, Kanagawa, Japan).

### 1.8 Statistical analysis

All data are expressed as mean ± SD. Statistical analysis was carried out via two-way analysis of variances (ANOVA) tests using SPSS software (SPSS Inc, Chicago, IL, USA). A value of *p* < .05 was considered as statistically significant; *p* < .01 was considered as very significant.

## 2. Results

### 2.1 Preparation of primary *w1/o*

In this study the primary *w1/o* was prepared by phase inversion composition (PIC) method, in which the emulsification is triggered by the change of composition. The optimum preparation process was screened and obtained with stirring speed 800–1000 rpm, emulsification temperature 60 °C and dispersing time 3 min. The *w1/o* primary emulsion was constituted by a blend of oil, LS (Span80:RH40, 10:8), co-LS and water phase.

The effects of oil and co-LS on the formation of primary emulsion were compared based on productivity, centrifuging stability after 5000 rpm for 5 min. The productivity of primary emulsion prepared by different oil phase followed the order of MCT > soybean oil > squalance > oleic acid. MCT, generally recognized as safe by the Food and Drug Administration (FDA), was selected as oil for further studies.

In addition, only Lecithin could help forming *w1/o* primary emulsion, which is transparent, translucent with blue opalescence. Thus, Lecithin (4%) was selected as co-LS.

### 2.2 Preparation of *w1/o/w2*

The *w1/o/w2* was prepared by self-emulsification method. The primary *w1/o* emulsions were mixed with HS and co-HS, the *w1/o/w2* MMEs would be formed in aqueous phase under mild stirring. Various formulations of coupled HS/co-HS were prepared according to L16（4^2^）orthogonal design (Table 1). The influences of HS and co-HS on the formation of MMEs are shown in Table 2. The results were analyzed by direct observation. RH40 exhibited best score compared with other HSs considering Y1, Y2 and Y3. The overall scores of glycerol and lecithin are higher than propylene glycol. The self-emulsifying efficiency of glycerol was higher than others, but the productivity of lecithin is highest. Lecithin was selected as co-HS finally.

**Table 2.** Results of orthogonal experiment design.

No.	HS	Co-HS	Y1	Y2	Y3	Overall score
1	RH40	Glycerol	5	60	5	27
2	RH40	Lecithin	4	68	5	29.9
3	RH40	Propylene glycol	4	40	1	17.5
4	RH40	/	5	50	5	23
5	EL30	Glycerol	5	45	5	21
6	EL30	Lecithin	4	54	5	24.3
7	EL30	Propylene glycol	3	30	1	13.2
8	EL30	/	4	28	5	13.9
9	RH40/Span80	Glycerol	4	53	5	23.9
10	RH40/Span80	Lecithin	4	55	5	24.7
11	RH40/Span80	Propylene glycol	4	35	1	15.5
12	RH40/Span80	/	4	38	3	17.3
13	EL30/Span80	Glycerol	4	27	3	12.9
14	EL30/Span80	Lecithin	4	30	3	14.1
15	EL30/Span80	Propylene glycol	2	15	1	6.9
16	EL30/Span80	/	2	13	3	6.7

**Figure 1.**
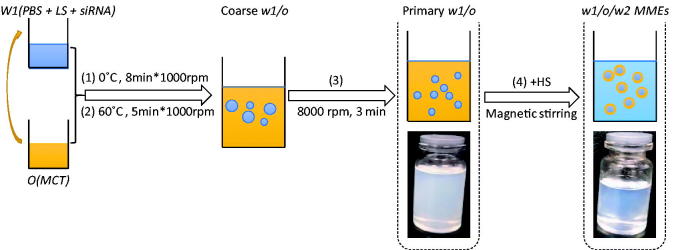
The preparation flowchart of MMEs.

The *w1/o* primary emulsion was constituted by a blend of 75% MCT, a 9–12% LS mixture composed of span80 and RH40 (10:8), 3–4% co-LS Lecithin and a 10–12% water phase. The HSs were a mixture of RH40/Lecithin (8:2). The final self-emulsifying MMEs were obtained by the mixture of *w1/o* primary emulsion with HSs with a weight ratio 4:1. The preparation flowchart could be described as Figure 1.

### 2.3 Characterization of self-emulsifying MMEs

**Figure 2.**
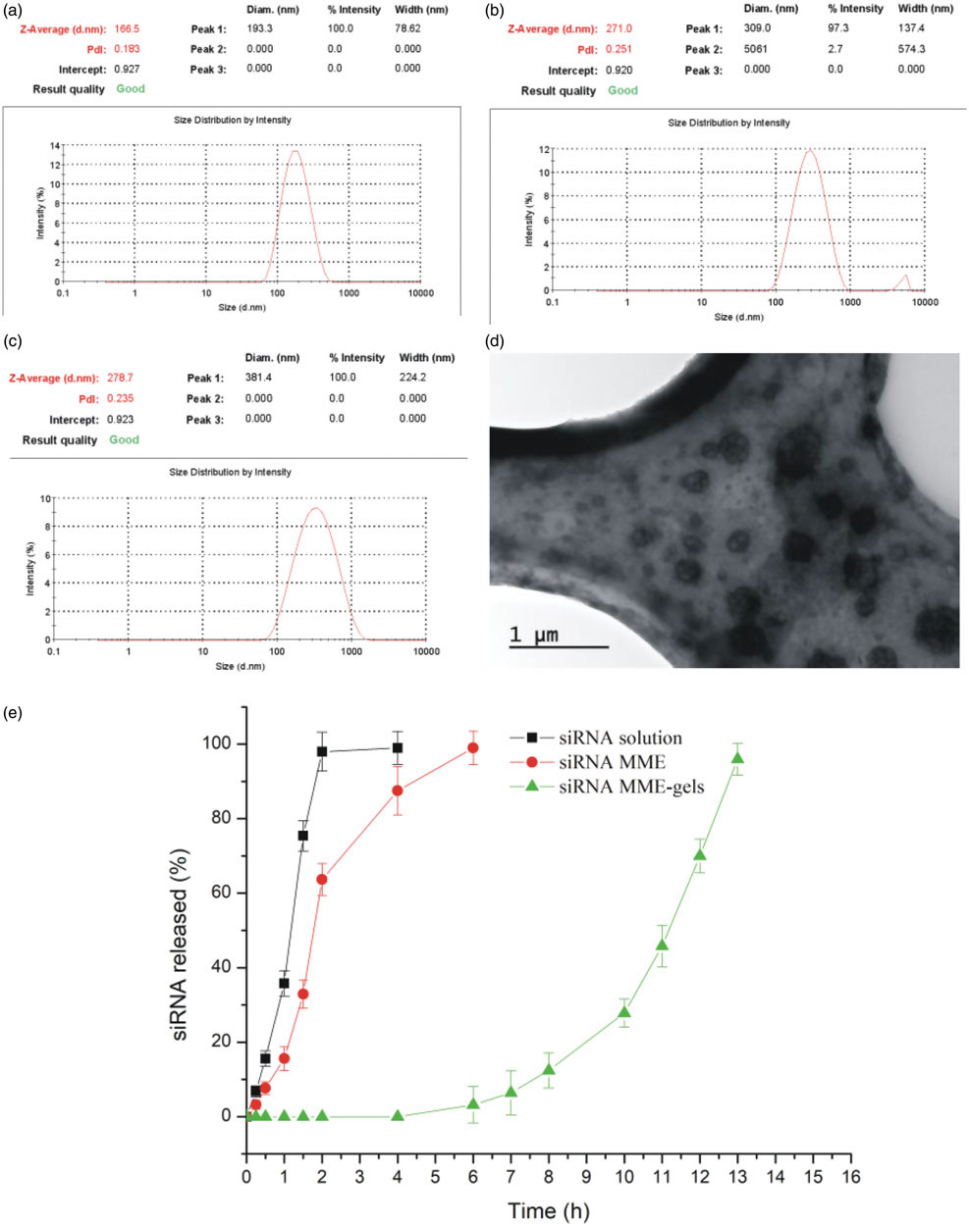
(a–c) Size of MMEs in dH_2_O, PBS and culture medium respectively (*n* = 3); (d) typical TEM micrograph of MMEs in PBS; (e) dissolution profiles of siRNA from MME, MME-gels and solution (*n* = 3).

As shown in Table 3, the *w1/o/w2* MMEs could be formed by mild agitation or shaking before use in dH_2_O, PBS and culture medium with size ranged from 166.5 to 278.7 nm (shown in Figure 2(a–c). The particle size of MMEs in dH_2_O is smallest, while the zeta potential is negative. Typical TEM micrograph of MMEs were shown in Figure 2(d), which indicated the particles of microemulsion were well regulated.

**Table 3.** Self-emulsifying ability comparisons in different solvents.

	dH_2_O	PBS	Culture medium
Emulsification	Translucent solution with blue opalescence	Milky-white fine emulsions, with blue opalescence after shaking	Red fine emulsions
Diameter/nm	166.5 ± 13.1	271.0 ± 11.1	278.7 ± 12.1
Zeta potential/mV	−7.43 ± 1.84	2.32 ± 0.10	0.15 ± 0.09
PDI	0.183 ± 0.040	0.251 ± 0.061	0.235 ± 0.051

The release behaviors of both siRNA solution and siRNA MME-GELs are illustrated in Figure 2(e). As for the control group, the siRNA could be detected within 0.25 h. The accumulative release percent soon rose to 95% after 2 h of incubation. On the contrary, siRNA couldn’t be detected within 5 h in the MME-gels group. 5 h time-lag was probably attributed to the diffusion of siRNA from internal water phase *w1* to the outer water phase *w2* and retardant effects of gels with high viscosities. After 11 h, the accumulative release percent increase to 70%. The *in vitro* release results in this study demonstrated that MME-GELs can be considered as a matrix system allowing a sustained delivery of siRNA.

### 2.4 Cytotoxicity evaluations of MMEs

**Figure 3.**
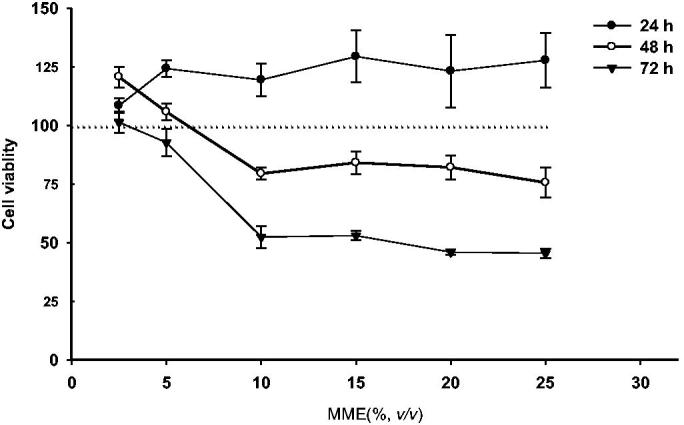
Cytotoxic activities of MMEs on *Hela* cells after 24 h, 48 h and 72 h of treatment. Data were expressed as mean ± SD (*n* = 5).

In this study, we evaluated the cytotoxicity of *w1/o/w2* MMEs in *Hela* cells by WST-8 method at varying time points. As indicated in Figure 3, the viability of *Hela* cells was nearly 100%, and none of treatment groups had any significant cytotoxicity compared to the negative control group for up to 24 h, which can prove that MMEs exhibit low cytotoxicity. However, the cytotoxicity of MMEs increased with the amount in a time dependent way. After 24 h of incubation, there was some slight reduction in cell viability when the concentration of MMEs rose to 0.10 μL/μL (%, *v/v*). The concentration of MMEs inhibiting 50% cell viability (IC50) was determined as IC50 = 0.166 μL/μL (16.6%, *v/v*) at 72 h.

### 2.5 Cellular uptake studies of MMEs in *Hela* cells

**Figure 4.**
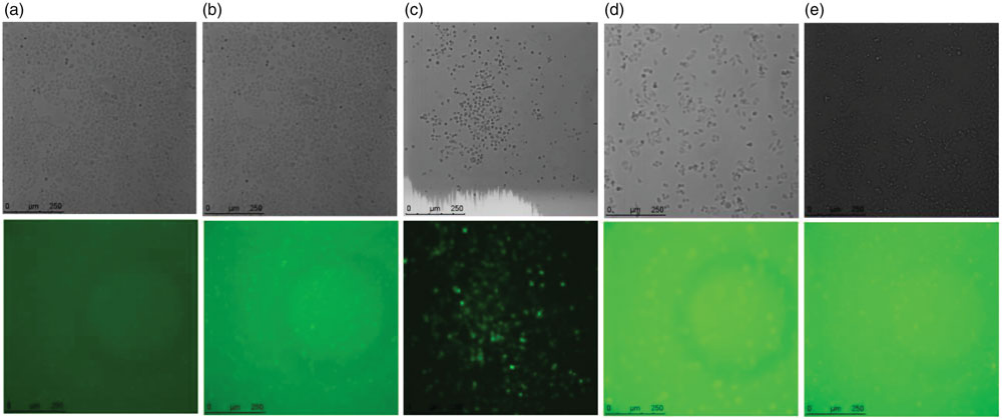
Cellular uptake efficiency *in Hela* cells. Representative fluorescence images of *Hela* cells treatment with (a) pure FAM-siRNA; (b) FAM-siRNA-Lipo2000; (c) FAM-siRNA-Lipo2000 + gels; (d) FAM-siRNA-MMEs; (e) FAM-siRNA-MMEs + gels.

The qualitative cellular uptake of *w1/o/w2* MMEs in *Hela* cells was investigated by treatment with FAM-siRNA-MMEs as compared with transfection reagent Lipo2000 by fluorescence microscope. Intracellular uptake was analyzed at 6 h time after incubation of labeled siRNA particles at a siRNA concentration of 0.27 μg/mL. Representative fluorescence images of *Hela* cells treatment with different formulations were shown and compared in Figure 4. Higher siRNA cellular uptake of FAM-siRNA-MMEs (column **d**) was observed in comparison with cells treated with negative control (column **a**) and Lipo2000 (column **b**) after 6 h of treatment through nonspecific endocytosis, in agreement with earlier reports on this formulation.

### 2.6 Gene silencing in *Hela* cells and mouse vaginal mucosa

**Figure 5.**
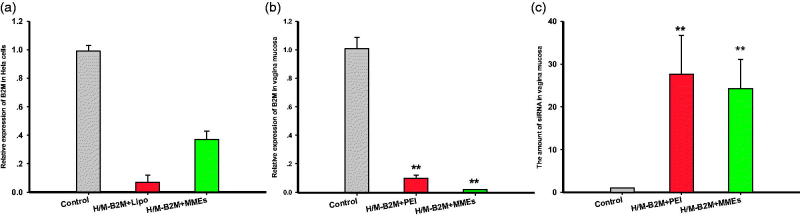
Relative B2M expression in (a) *Hela* cells and (b) vaginal mucosa treatment with different formulations. (c) The amount of siRNA in vaginal mucosa. Data were expressed as mean ± SD (*n* = 6). *p < .05; **p < .01.

The efficiency of resultant siRNA-MMEs and siRNA-Lipo2000 to cause gene silencing in *Hela* cells in the female reproductive tract were investigated meantime at concentration of 0.27 μg/mL, with random Oligonucleotides-Lipo2000 (NC). As shown in Figure 5(a), siRNA-MMEs could transfect siRNA into cells and inhibit the expression of B2M target gene with very high efficiency. Commercial transfection agent Lipo2000 acted as positive control seemed to show more potent and produced better gene silencing (0.07 ± 0.05, *p* < .01) than siRNA-MMEs (0.37 ± 0.06, *p* < .05) in comparison with NC (0.99 ± 0.04).

Interestingly, the efficiency of siRNA-MMEs-gels to cause gene silencing in vaginal mucosa was comparable (0.0189 ± 0.00129) or even better than conventional transfection agent PEI (0.0965 ± 0.0229) at concentration of 127.22 μg/mL compared with random oligonucleotide-PEI (1.00 ± 0.0778, shown in Figure 5(b)), which in accordance with the siB2M quantity (shown in Figure 5(c)). The amount of siRNA in vagina mucosa were determined as 24.2 ± 6.79 (*p* < .01) and 27.6 ± 9.03 (*p* < .01), much higher than NC (1.01 ± 0.03).

### 2.7 Histological analyses

**Figure 6.**
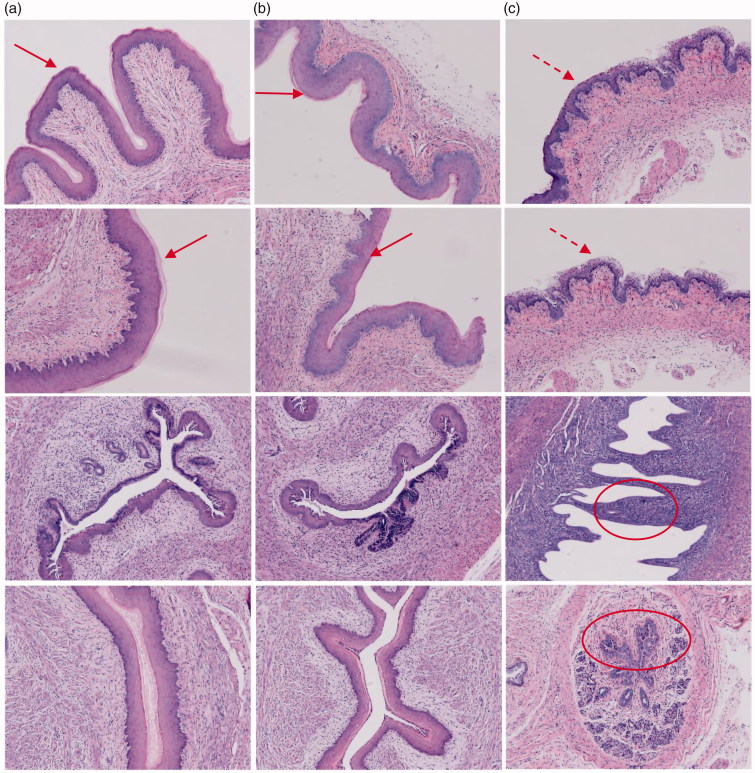
Histopathology of vaginal tissue in mice instilled with PBS (a), MMEs-gels (b) and PEI-gels(c).

As shown in Figure 6, histologic analysis of tissue sections from the vagina in mice treated with PBS (a) or MMEs-gels(b) showed minimal to no inflammation by haematoxylin eosin (HE) staining, exhibited intact mucosal layer and similar vagina epithelial thickness (a, b arrows), and showed no significant pathologic findings (Woodrow et al., 2009; Li et al., 2012). However, PEI-gels treated mice had barely mucosal layer and marked vaginal epithelial hyperplasia(c dotted arrow) and frequent infiltrating neutrophils within the lumen (c ovals) indicative of a mild to moderate vaginitis.

## 3. Discussion

In this study, we developed a simple alternative delivery strategy for siRNA based on thermosensitive gels and MMEs. We showed that siRNA can be densely encapsulated within MMEs and when applied topically to the vaginal mucosa, lead to highly efficient gene silencing.

The two-step emulsification process able to produce MMEs generally requires high thermal and mechanical energy input, which limits their application to thermo-resistant drugs. An alternative way is the application of low-energy method, which uses the internal chemical energy of the system to achieve emulsification. The low-energy methods could be classified depending on whether or not changes in the surfactant spontaneous curvature happened during the preparation. When changes in the surfactant spontaneous curvature are produced they are designated as ‘phase inversion’ methods, including phase inversion temperature (PIT) and phase inversion composition (PIC) methods if emulsification is triggered by a change in temperature or composition, respectively (Solans & Sol, 2012). In this study the primary *w1/o* was produced by PIC method. On the other side, nano-emulsion formation triggered by the rapid diffusion of surfactant and/or solvent molecules from the dispersed phase to the continuous phase without involving a change in the spontaneous curvature of the surfactant is referred to as ‘self-emulsification’, as was employed in the preparation of *w1/o/w2* MMEs in this study.

The stability and productivity of *w1/o* emulsion related closely with the physicochemical properties of oil such as molecular volume, chain length and viscosity (Sadurni et al., 2005; Matos et al., 2014). Moreover, the biological safety should also be considered seriously. Oil should interact with interfacial molecules(surfactants) in some way: the smaller of the molecular volume is, the better of the compatibility would be. Molecular chain length should be in a suitable range to form microemulsion, a prolonged chain length is not preferred. The viscosity of oil should be in the range of 0.05–0.12 Pas to achieve appropriate thickness of w/o interfacial film: if it’s too thin, the *w/o* emulsion is not stable; otherwise may increase the particle size, the amount of surfactants and plasticity of emulsion.

The *w1/o/w2* was prepared by self-emulsification method in this study. It has been established that during second step of double emulsion emulsification, the applied shearing force might disrupt the primary emulsion droplets, thus the inner aqueous phase containing siRNA mixed with outer aqueous phase, allowing diffusion of siRNA into outer aqueous phase, which may lead to poor loading efficiency, into outer aqueous phase (Schuch et al., 2014; Lei et al., 2016). The main drawback of the self-emulsifying method, in comparison with the high-energy methods to produce microemulsions, is the application of great amounts of synthetic surfactants, which may lead to toxic effects and hence limit its choice as a route of administration. In order to decrease the amount of single surfactant, the combination of different types of surfactants were extensively examined and compared. Mixed surfactants were considered to play synergistic effects in the preparation of *w1/o* primary emulsion and *w1/o/w2* MMEs. The combination of RH40(HLB 13–14) and Lecithin (HLB 4–5) was finally chosen as HS/co-HS probably due to the internal affinity and complementarity of HLB.

The size and zeta potential of MMEs were characterized in different mediums, such as dH_2_O, PBS and culture medium 1640. SiRNA was encapsulated into internal *w1* phase and supposed to shield from RNAase degradation. A sustained release was observed for siRNA from MMEs, which might probably be associated with molecular diffusion or reverse micelle transportation of siRNA from internal water phase driven by concentration gradient.

The cytotoxicity and mucosa irritation of MMEs were studied to evaluate the effects of mixed surfactants in the formulation of MMEs. The cytotoxicity tests were considered to be an effective and sensitive way to assess the harmful components of vectors. Compared with classical MTT method, WST-8 method was considered to be more accurate and could reduce the possibility of false positive results (Worle-Knirsch et al., 2006). We did not observe a decrease in cell viability of *HeLa* cells over the range of concentrations that were evaluated within 24 h.

Histological examination of HE stained sections of vaginal tissues from mice treated with PBS or MMEs were within normal limits and histologically similar. In contrast, mice treated with siRNA formulated into PEI had thickened vaginal epithelium with frequent foci of luminal polymorphonuclear neutrophils. These histological features are suggestive of symptoms associated with active vaginitis. From collaborative results of cytotoxicity and histological analyses, MMEs-gels showed good biocompatibility. In addition, our MMEs are less irritating and inflammatory compared to delivery of siRNA-PEI.

The cellular uptake of both MMEs and MMEs-gels were conducted in *Hela* cells, with Lipo2000 and Lipo2000-gels as positive controls. Lipo2000 exhibited more efficient cellular internalization than MMEs in *Hela* cells. The thermosensitive gels could assist the siRNA internalization delivered by both Lipo2000 and MMEs effectively, which is probably attributed to the prolonged contacting time with cells by cell adhesion, thereby promoting efficient interaction with the *Hela* cells membrane (Elzoghby, 2013; He et al., 2015). From the collaborative results of cytotoxicity and cellular uptake of MMEs, the enhanced cytotoxicity of *Hela* cells after 24 h might be probably attributed to enhanced cellular uptake as well as the increased intrinsic toxicity of MMEs.

Gene silencing efficiency of siRNA-MME and siRNA-MME-gels were observed in *Hela* cells and mouse vagina meantime, with siRNA-PEI and siRNA-PEI-gels as positive controls. Surprisingly, the increased gene silencing efficiency of siRNA delivered by MMEs was discovered in both *Hela* cells and mouse vagina than PEI, which might be related to several factors, such as the penetration enhancement of several surfactants and co-surfactants, the small size of the MMEs providing large interfacial surface area for siRNA absorption (Solans et al., 2005; Gutiérrez et al., 2008; Todosijević et al., 2015) and the prolonged vaginal residence time based on the mucoadhesion by gels (D'Cruz & Uckun, 2001; Andrews et al., 2009; Baloglu et al., 2009).

Thus, the construction of microgels by the self-emulsified MMEs formulation will provide higher mucosal residence time for higher exposure and lastly the intravaginal route of administration will provide direct access to the target tissue to show down-regulation of target gene in an experimental model established in mouse. The small RNA was encapsulated into the internal water phase as protection to the small RNA from the attack of RNAase, the *w1/o/w2* help the small RNA transport through the bio-membrane and increase the transfection efficiency. The efficacy of target gene silencing was attributed to better stability *in vitro* and *in vivo* and enhanced endocytosis. Our choice for multiple *w1/o/w2* microemulsion was orientated by the need to improve the topical therapeutically efficacy of HPV vaccines. The results showed that siRNA-MMEs with good emulsification property and fine globule size effectively suppressed the expression of target gene. The vaginal delivery of small interfering RNA (siRNA) to the vaginal mucosa may provide a promising therapeutic solution to a range of HPV infection disorders.
